# Occurrence of the S230R integrase strand inhibitor mutation in a treatment-naïve individual case report

**DOI:** 10.1097/MD.0000000000020915

**Published:** 2020-07-02

**Authors:** Smitha Gudipati, Indira Brar, Alicia Golembieski, Zachary Hanna, Norman Markowitz

**Affiliations:** Department of Infectious Disease, Henry Ford Hospital, Detroit, MI.

**Keywords:** case report, dolutegravir, integrase strand inhibitiors, next generation sequencing, resistance

## Abstract

**Rationale:**

Transmitted resistance to integrase strand inhibitors (INSTI) has been uncommon, but is slowly becoming more prevalent among those living with HIV. In an era with 2-drug regimens for antiretroviral therapy, transmitted resistance for INSTI is alarming.

**Patient concerns:**

A 28-year-old African American female was recently diagnosed with HIV during a 30-week prenatal visit.

**Diagnosis:**

HIV 4th generation test was positive as well as confirmation. Genotype was performed using next generation sequencing.

**Interventions:**

Patient was initially rapidly started on a dolutegravir based regimen and changed to a protease inhibitor regimen once her genotype reported an S230R mutation.

**Outcomes:**

Patient became virally suppressed on antiretroviral therapy and delivered an HIV negative baby.

**Lessons:**

INSTI resistance testing should be done for treatment-naïve and INSTI-naïve persons, particularly when considering 2 drug INSTI based regimens.

## 1 Introduction

Transmitted or pretreatment resistance to integrase strand inhibitors (INSTI) has been uncommon.^[1]^ A study from a North Carolina reference laboratory based on genotypes collected from November 2010 to September 2016, reported INSTI major resistance-associated mutations (RAMS) in 3 (0.37%) of 840 individuals who were diagnosed with HIV-1 infection within the prior 3 months.^[2]^ Samples from untreated individuals from sub-Saharan Africa revealed a 2.4% prevalence of major INSTI associated mutations (including S230R), all detected at a frequency threshold <20%.^[3]^ Modica et al reported 15% intermediate level of resistance to dolutegravir (DTG) in their population of INSTI-failing patients, suggesting that a large reservoir exists for transmission of INSTI RAMS.^[4]^ Among our patients, we have observed INSTI RAMS (detection frequency ≥1%) in 0.2% of 229 treatment-naïve and 7% of 293 INSTI-experienced individuals.^[5]^ Here, we describe a case of pretreatment resistance in a patient newly diagnosed with HIV-1-infection found to have the accessory S230R mutation before initiating DTG, emtricitabine (FTC), and tenofovir disoproxil fumarate (TDF).

## 2 Case report

This study was exempt from the Henry Ford Hospital Institutional Review Board approval as it is a case report on a single patient, and the patient gave verbal consent for publication.

A 28-year-old African American female with no significant past medical history was diagnosed with HIV infection during a 30-week prenatal visit. She had no prior pregnancies and had never been HIV tested before this visit. The patient had not sought prenatal care earlier in the pregnancy due to insurance issues. Her CD4 count at the time of diagnosis was 481 cells/mm^3^ and viral load was 1030 copies/mL. The patient was started on DTG, FTC, and TDF the day diagnosis was confirmed and a genotype (performed by next-generation sequencing) was drawn before treatment initiation. The genotype resulted at day 15 after antiretroviral therapy was initiated and revealed clade B HIV-1 with G163R and S230R INSTI mutations, the latter present at a detection frequency of 16.2%, reported as conferring low-level DTG resistance.^[6]^ There were no other significant drug resistance mutations in the reverse transcriptase or protease genes, and the virus was susceptible to TDF and FTC. Her viral load was undetectable at day 15. Despite viral suppression, the patient was switched to a regimen of darunavir and ritonavir twice daily with FTC and TDF. She maintained an undetectable HIV viral load and delivered an HIV negative baby.

## 3 Discussion

Resistance to second generation INSTI although rare, can occur and has been reported to be a cause of inadequate response to antiretroviral therapy in patients.^[7]^ Known risk factors to DTG resistance include infection with a non-B subtype of HIV-1, a high viral load, low CD4 cell count,^[8]^ and insufficient adherence to antiretroviral therapy.^[9]^ Common INSTI RAMS include R263K, Q148H/R/K, G118R, G140A/S/C, E138A/K/T, N155H, and Y143C/R.^[7]^ This report presents a case of an accessory mutation, linked to DTG resistance, likely acquired by transmission from the patient's HIV infected partner.

The S230R mutation has been previously described in 2 individuals failing DTG monotherapy in the DOMONO study.^[10,11]^ Pham et al, using an infectious molecular clone with the insertion of S230R by site-directed mutagenesis, showed that this mutation conferred a 63% reduction of integrase enzyme efficiency and a fold change in mean IC_50_ of 3.85, 3.72, 1.52, and 1.21 for DTG, cabotegravir, raltegravir. and elvitegravir (EVG), respectively, compared to virus lacking S230R.^[6,12]^ These results demonstrated that the S230R substitution caused similar effects on viral replicative capacity as R263K,^[12]^ which is known to be selected in vitro by EVG, DTG, and BIC causing viral resistance on an incompletely suppressive DTG containing regimen.^[13]^ Phenotypic analysis of this patient's virus found IC_50_ fold changes of 0.86, 0.87, 1.30, and 0.89 to bictegravir, DTG, EVG, and raltegravir, respectively. This lack of detectable phenotypic resistance may have been reflective of the low copy number of virus carrying the S230R mutation (167 copies/mL).

Two drug regimens are now potential options for treatment-naïve individuals. These regimens are being considered as standard of care antiretroviral therapy given the concerns for toxicities from the medications that are being taken for longer duration as our patient population is aging.^[14]^ Both the GEMINI-1 and GEMINI-2 studies reported noninferior efficacy and similar tolerability profile of DTG plus lamivudine to a guideline recommended 3-drug regimen at 48 weeks in newly diagnosed HIV patients.^[15]^ Although these results seem promising, given reports of DTG acquired resistance in naïve patients, we propose that INSTI resistance testing be performed before initiating therapy, especially if the patient is to be started on a 2 drug INSTI based regimen.

Our report has a few limitations. We were unable to obtain partner's virus for analysis to determine if they also had the S230R mutation as well. Additionally, although our patient exhibited an initial response to INSTI based antiretroviral therapy, the long-term impact of this mutation on virologic outcome has not been fully elucidated. The patient's regimen was switched despite virological suppression given the concern of potential treatment failure and the risk of maternal-child transmission.

In conclusion, this report adds to mounting evidence that INSTI resistance testing be done for treatment-naïve and INSTI-naïve persons, particularly when considering 2 drug INSTI based regimens.

## Author contributions

Smitha Gudipati: Contributed as primary author of writing the case report.

Indira Brar: Made intellectual contributions to the design and analysis of the study.

Alicia Golembieski: Contributed by performing the genotype assay for the patient.

Zachary Hanna: Made intellectual contributions to the design and analysis of the study.

Norman Markowitz: Made intellectual contributions to the design and analysis of the study as well as contributed by performing the genotype assay for the patient.
